# Entomological Adaptive Sampling Framework (EASF) for malaria vector surveillance: an economic evaluation of pilot implementation in Ghana and Mozambique

**DOI:** 10.1186/s12936-026-06020-w

**Published:** 2026-07-13

**Authors:** Anton L. V. Avanceña, Nicholas W. Daniel, Mercy Opiyo, Steven Gowelo, Allison Tatarsky, Luis Jamu, Dulcisária Marrenjo, Samuel K. Oppong, Ernest Boampong, Christian Atta-Obeng, Otubea Owusu-Akrofi, Keziah Laurencia Malm, Neil Lobo, Edward Thomsen

**Affiliations:** 1 Health Outcomes Division, College of Pharmacy, The University of Texas at Austin, 2409 University Ave, PHR 2.112, Austin, TX 78712 USA; 2 Department of Internal Medicine, Dell Medical School, The University of Texas at Austin, Austin, TX USA; 3 Department of Biological Sciences, University of Notre Dame, Notre Dame, IN USA; 4 Malaria Elimination Initiative, Institute for Global Health Sciences, University of California, San Francisco, San Francisco, CA USA; 5 Centro de Investigação em Saúde de Manhiça (CISM), Maputo, Mozambique; 6 Programa Nacional de Controlo da Malaria, Ministry of Health, Maputo, Mozambique; 7 National Malaria Elimination Programme, Public Health Division, Ghana Health Service, Accra, Ghana; 8 School of Population Health, Curtin University, Bentley, WA Australia; 9 The Center for Evolution & Medicine, School of Life Sciences, Arizona State University, Tempe, AZ USA

**Keywords:** Entomological surveillance, Adaptive sampling, Economic evaluation, Cost-effectiveness

## Abstract

**Background:**

Entomological surveillance provides critical information for National Malaria Programs (NMPs), yet there is limited evidence on the cost-effectiveness of alternative sampling approaches. Conventional surveillance often relies on purposive, fixed-site sampling, which may produce biased or non-representative data. To address these limitations, the Entomological Adaptive Sampling Framework (EASF) was developed to optimize sampling strategies based on spatiotemporal vector dynamics. This study evaluates the costs and cost-effectiveness of EASF compared to routine entomological surveillance in Ghana and Mozambique.

**Methods:**

We conducted a cost and cost-effectiveness analysis of EASF using prospective data from pilot implementations in Ghana (July 2023–2024) and Mozambique (September 2022–August 2024). Costs (in current US$) were estimated from a payer perspective through microcosting , categorized by input type and activity. Effectiveness was measured based on collection yields, namely the total number of mosquitoes collected and the average number of mosquitoes per collection. Incremental cost-effectiveness ratios (ICERs) were calculated to compare EASF with routine entomological surveillance.

**Results:**

The total costs of EASF were $144,688 in Ghana (13 months) and $48,646 in Mozambique (9 months). EASF was associated with higher costs than routine surveillance ($90,004 in Ghana; $22,311 in Mozambique). On average, EASF was associated with 0.75 and 1.12 more mosquitoes per collection in Ghana and Mozambique, respectively. The unit cost of each mosquito collected was lower under EASF ($11.10 vs. $43.65 in Ghana; $54.23 vs. $743.69 in Mozambique). The ICERs of EASF were $4.98 and $30.38 per additional mosquito collected, and $30.23 and $72.76 per increase in the average number of mosquitoes per collection in Ghana and Mozambique, respectively.

**Conclusions:**

EASF has the potential to improve the efficiency of entomological surveillance by collecting more mosquitoes at lower costs. Future research should build on these findings by demonstrating the downstream value of improved entomological data on NMP decision-making and malaria transmission. A budget impact analysis should assess the affordability of scaling up EASF.

**Supplementary Information:**

The online version contains supplementary material available at https://doi.org/10.1186/s12936-026-06020-w.

## Introduction

Despite the importance of entomological surveillance in malaria control and elimination, the most cost-effective or optimal entomological sampling technique is unknown. There is no scientifically grounded guidance on the number and location of sampling sites, sampling frequency, and sample size to produce accurate and representative data that may be used by national malaria programs (NMPs) to guide programmatic and strategic decisions. The World Health Organization (WHO) recommends stratified sampling, which involves purposive selection of sites by eco-epidemiological zones within a given country based on historical disease foci or local knowledge and/or selected based on convenience and accessibility [1]. However, purposive sampling may produce surveillance data that is neither tailored to programmatic objectives nor generalizable for decision-making and resource allocation [2]. Additionally, static, fixed, or non-adaptive sampling methods that do not reflect dynamic local conditions or contexts (e.g., changes in vector populations or interventions over time and space) may lead to biased data.

The Entomological Adaptive Sampling Framework (EASF) was developed to address the limitations of conventional entomological surveillance that relies on fixed sentinel sites. EASF uses a Bayesian, spatiotemporal model that informs adaptive sampling strategies, such as varying site locations and frequency of trappings, based on malaria transmission dynamics over time. The model combines data on historical *Anopheles* abundance and environmental variables derived from remote sensing. An optimization process, described in a previous report [3], prioritizes areas with higher predicted mosquito abundance and greater uncertainty, thereby targeting potential transmission hotspots. By optimizing the number or frequency of collections, the EASF algorithm suggests precise locations (e.g., villages or regions) that will yield the highest (predicted) number of mosquitoes collected. Research and field teams then select nearby suitable and accessible locations in consultation with local communities. The effectiveness of EASF was assessed through pilot implementations in Mozambique and Ghana. These implementations found that sampling from EASF-suggested sites resulted in more mosquitoes collected, higher robustness of the resulting models, greater predictive ability, reduced uncertainty, and greater efficiency in targeting informative sites compared to standard sentinel sites [3].

While EASF was found to achieve better entomological surveillance outputs, its adoption and scale-up will also depend on its economic value or “value for money” relative to current surveillance approaches. In this study, we conducted cost and cost-effectiveness analyses of EASF based on pilot implementation in Ghana and Mozambique. Results from this economic evaluation can inform decisions by NMPs to adopt EASF. With a limited body of economic research on entomological surveillance, this study also provides a framework for understanding the cost and cost-effectiveness of novel entomological surveillance approaches.

## Methods

### Overview

Using data from pilot implementations in Ghana and Mozambique [3], this study estimated the cost and cost-effectiveness of EASF compared to routine entomological surveillance. In the cost analysis, we estimated and compared the cost of EASF and routine entomological surveillance based on the resources used to implement each strategy. We adopted a payer perspective, which means that all costs incurred by malaria program funders (e.g., national and local governments, donors, private sector, etc.) were estimated. In the cost-effectiveness analysis, we estimated the incremental (i.e., additional or extra) cost per unit of incremental effectiveness or output of EASF compared to routine entomological surveillance. The effectiveness of each strategy was measured in terms of entomological surveillance outputs, namely the total number of mosquitoes collected, an optimization criterion in the EASF algorithm [3], and the average number of mosquitoes per collection. These outputs are important for NMPs looking to improve the efficiency and quality of their entomological surveillance, which informs their programmatic decisions, such as the subnational allocation of bednets or insecticide spraying [4–7]. Costs and effectiveness were not discounted due to the short analytic period. We followed guidelines in economic evaluation, including the Consolidated Health Economic Evaluation Reporting Standards and reference cases for economic evaluation of global health interventions [8–10]. Analyses were conducted in R version 4.5.0 (R Foundation for Statistical Computing, Vienna, Austria).

### Data sources

Data for this study were prospectively collected during pilot implementations of EASF in Ghana and Mozambique (Additional file 1), described elsewhere [3]. In summary, EASF was piloted in Mozambique from September 2022 to August 2024 (24 months total) in 2 provinces, Niassa (Cuamba and Mandimba Districts) and Zambezia (Gurué and Morrumbala Districts). In year 2 of the EASF implementation, Gurué District was dropped due to limited resources, and only its year 1 costs were included in the analysis. In Ghana, the EASF pilot took place from July 2023 to July 2024 (13 months total) in 3 regions and 5 districts: Greater Accra (Ga West & Ga South), Volta (Ketu South & Ketu North), and Western North (Suaman). Routine entomological surveillance, to which EASF was compared, was implemented in the same regions in each country, though the periods of data collection varied. In Ghana, routine entomological surveillance overlapped with EASF from July 2023 to July 2024 (13 months total). In Mozambique, data on the costs of routine entomological surveillance were only collected in Cuamba and Mandimba from December 2023 to August 2024 (9 months total; Additional file 1).

EASF and routine entomological surveillance in both countries were centrally managed by the respective NMPs. In Ghana, supervisory visits to sampling sites were conducted by central and regional NMP personnel, monthly in EASF districts and quarterly in routine districts. In Mozambique, coordination was managed centrally, while implementation and supervision at the district level were led by NMP provincial entomologists. Mosquito collection methods for EASF and routine entomological surveillance were standardized within each country. In Ghana, collections used human landing catches (HLC) and pyrethrum spray catches (PSC), while collections in Mozambique used HLC, Centers for Disease Control and Prevention (CDC) light traps (LTs; Model 512, John W. Hock Company, Florida, USA) either alone or combined with tents, and Prokopack aspirators (12 V, Model 1419, John W. Hock Company, Florida, USA). Further details about the trapping in Mozambique and Ghana are described in a separate report [3].

#### Cost data

Cost data for EASF and routine entomological surveillance were collected through expenditure and financial records maintained by implementing partners in each country. EASF activities that incurred costs included trainings with NMP and subnational staff, mosquito collection efforts, and supervision and monitoring visits. We excluded personnel costs associated with central and subnational (i.e., provincial and district) NMP staff, which were assumed to be the same for EASF and routine entomological surveillance. We also excluded the costs of developing and running the dynamic statistical model for EASF that predicts the optimal sampling locations. In a sensitivity analysis, we included the start-up costs of the EASF model, estimated at US$150,000 or $75,000 per country. All costs were in current US$.

#### Effectiveness data

Data on the effectiveness of EASF and routine entomological surveillance were based on outputs collected and reported by entomological staff during implementation, including the sampling effort or number of collections, the number of mosquitoes collected, and the average number of mosquitoes per collection (Table 1). In Ghana, data collection involved a hybrid approach, where field staff initially recorded data on paper forms, which were then digitized and transferred onto the District Health Information Management System 2 for centralized management and analysis. In Mozambique, data was initially collected using paper forms, which were later digitized and transferred to Microsoft Excel for data cleaning and analysis. All household locations where sampling was conducted were georeferenced using the Global Positioning System.

**Table 1:** Entomological outputs of EASF and routine entomological surveillance in Ghana and Mozambique

Output	EASF	Routine	Incremental effectiveness^a^
Ghana			
Collections			
Total	2996	572	2424
Ave. per month	230	44	186
HLC (% of total)	947 (32)	227 (40)	720
PSC (% of total)	2049 (68)	345 (60)	1704
Mosquitoes			
Total	13,036	2062	10,974
Ave. per collection	4.35	3.60	0.75
Mozambique^b^			
Collections			
Total	756	432	324
Ave. per month	84	48	36
HLC (% of total)	756 (100)	432 (100)	324
Mosquitoes			
Total	897	30	867
Ave. per collection	1.19	0.07	1.12

^a^Incremental effectiveness was calculated by subtracting the outputs from EASF by the outputs from routine entomological surveillance

^b^Results are limited to Cuamba and Mandimba, where cost and entomological data for EASF and routine entomological surveillance were collected for the same 9-month period (December 2023–August 2024)

*EASF* Entomological Adaptive Sampling Framework; *HLC* human landing catch; *PSC* pyrethrum spray catch.

### Analysis

#### Cost analysis

Using a top-down microcosting or ingredients-based approach, we calculated the costs of EASF and routine entomological surveillance separately for Ghana and Mozambique [11]. Costs were labeled by type of expense (e.g., transportation, office supplies, per diems) and function or activity (e.g., supervision, collection, monitoring). Costs were also grouped by input or resource (personnel, consumables, capital, and services; Table 2). Per diems for current staff at the national and subnational levels were classified as service costs, while per diems for community volunteers (who performed collections using HCL and CDC light traps) and supervisors engaged specifically for EASF were classified as personnel costs. In addition to cumulative or total costs, we calculated costs per month and by district.

**Table 2:**
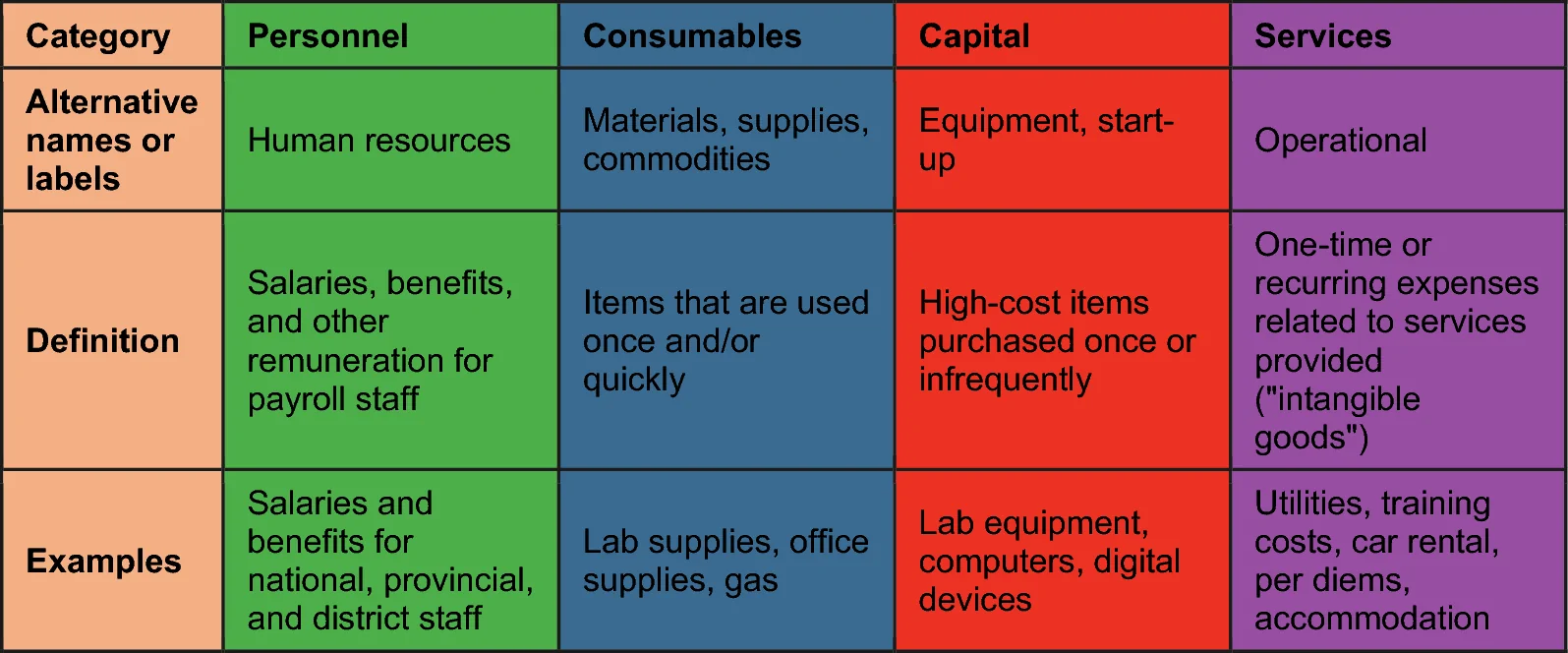
Main and subcategories of costs

The incremental cost of EASF was calculated by subtracting its total cost from the total cost of routine entomological surveillance. For Mozambique, we conducted additional adjustments to account for the shorter data collection period (Additional file 1) for routine entomological surveillance (December 2023-August 2024; 9 months) compared to EASF (September 2022-August 2024; 24 months). We first calculated the average monthly cost of EASF by dividing its total cost by 24 months. This “standardization” or “smoothing” of EASF costs captures cost fluctuations over the entire implementation period, such as year 1 training costs. We then multiplied the average monthly cost of EASF by 9 to ensure that the total costs were comparable to those of routine entomological surveillance.

To understand the relative efficiency of each surveillance approach, we also calculated the unit costs of EASF and routine entomological surveillance by dividing the total cost by total outputs. Specifically, we calculated the costs per collection and mosquito collected. This approach is analogous to comparing each approach to a null or no-intervention scenario, which is aligned with the WHO’s generalized cost-effectiveness analysis methodology [12].

#### Cost-effectiveness analysis

The main measure of cost-effectiveness is the incremental cost-effectiveness ratio (ICER; Eq. 1), calculated by dividing the incremental costs (ΔCosts) and incremental effectiveness or outputs (ΔEffectiveness) of two competing alternatives (a and b). The ICER represents the additional cost of one unit of output—measured in this study as the number of mosquitoes collected and the average number of mosquitoes per collection—of a new strategy compared to the existing alternative.

1 $$ICER = \frac{\Delta Costs}{{\Delta Effectiveness}} = \frac{{C_{b} - C_{a} }}{{E_{b} - E_{a} }}$$

## Results

### Cost analysis of EASF

**Fig. 1:**
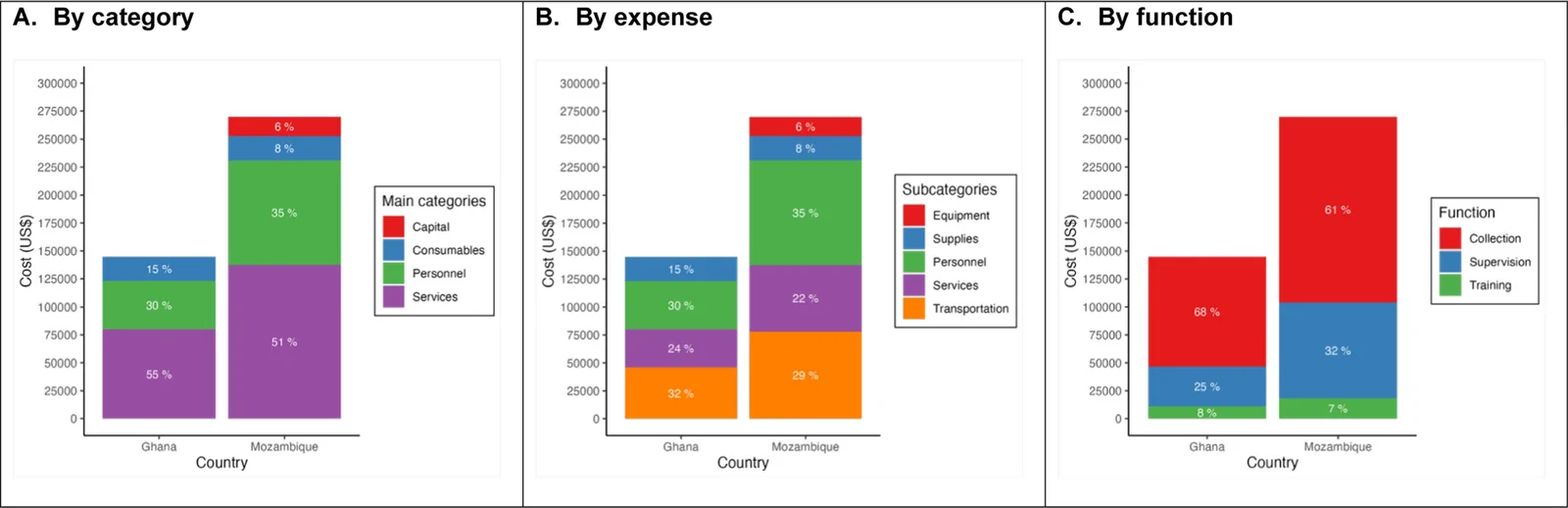
Total costs of EASF by category, expense, and function in Ghana and Mozambique. These bar graphs show the distribution of costs by category (**A**), expense (**B**), and function (**C**). *EASF* Entomological Adaptive Sampling Framework

The total costs of the EASF pilot implementation in Ghana was $144,688 over 13 months for 5 districts (Ketu South, Ketu North, Suaman, Ga West, and Ga South). In Mozambique, the total costs of EASF pilot implementation were $269,903 over 24 months for 4 districts (Gurué [year 1 only], Cuamba, Mandimba, and Morrumbala Districts). In both countries, the largest share of costs was for services such as staff per diems and transportation costs (55% or $80,004 in Ghana and 51% or $137,689 in Mozambique), followed by personnel (30% or $43,162 in Ghana and 35% or $93,274 in Mozambique; Fig. 1A). By type of expense, a total of 86% of costs in both countries were incurred for transportation (32% or $45,830 in Ghana and 29% or $77,939 in Mozambique), personnel (30% or $43,162 in Ghana and 35% or $93,274 in Mozambique), and service-related (24% or $34,173 in Ghana and 22% or $59,751 in Mozambique) expenses (Fig. 1B). By function, about two-thirds of the costs went to collections (68% or $98,061 in Ghana and 61% or $165,891 in Mozambique), while a smaller share was allocated to supervision (25% or $35,483 in Ghana and 32% or $85,716 in Mozambique), followed by one-time training costs (8% or $11,145 in Ghana and 7% or $18,296 in Mozambique; Fig. 1C).

**Fig. 2:**
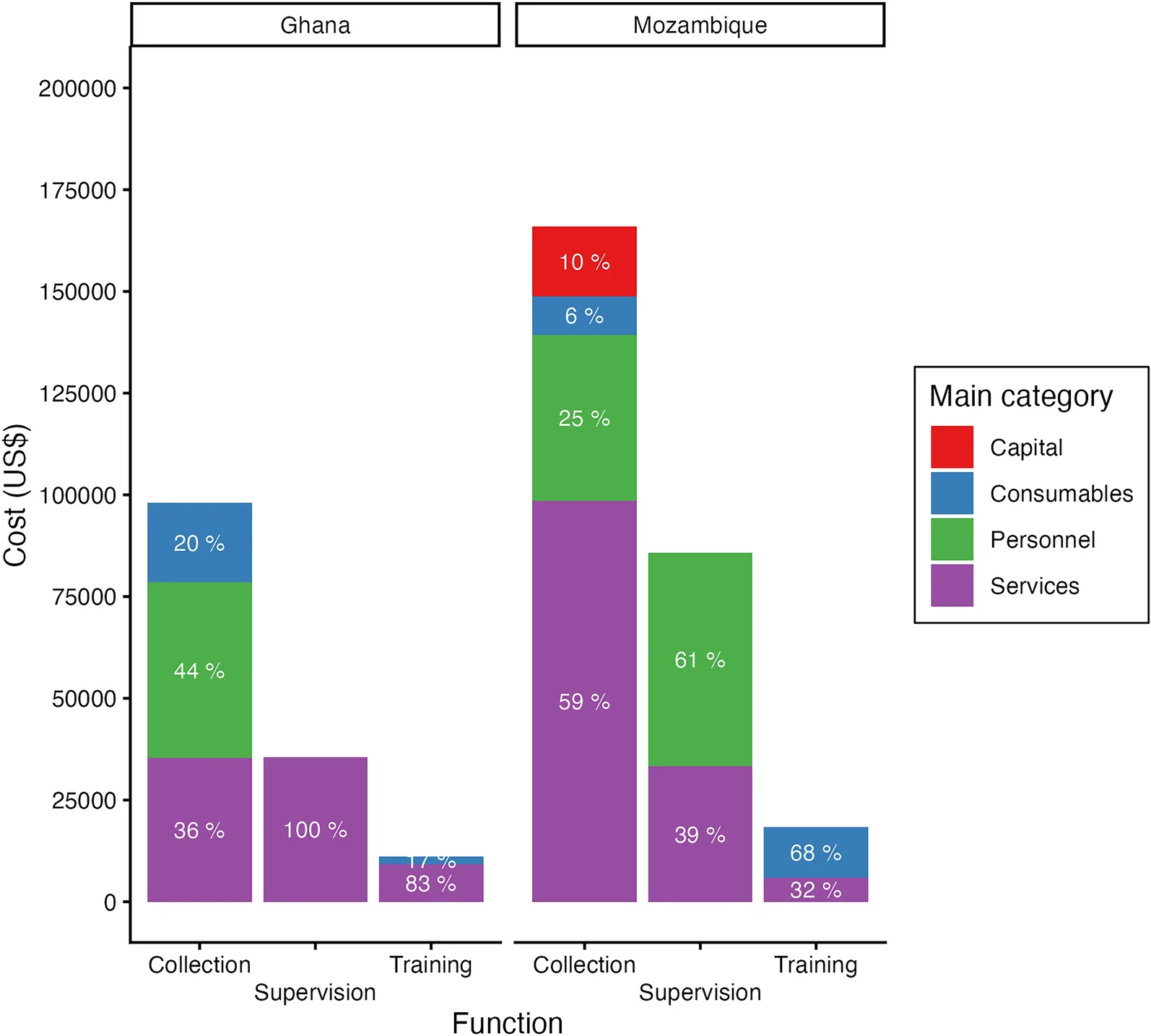
Total costs of EASF by category and function in Ghana and Mozambique. These bar charts show the proportion of total costs by category (capital, consumables, personnel, and services) and function (collection, supervision, and training). Cost data collection occurred over 13 months in Ghana and 24 months in Mozambique, though the length of data collection varied by district. *EASF* Entomological Adaptive Sampling Framework

We found differences in resource use when examining costs by function and category (Fig. 2). In Ghana, services were 83% and 100% of training and supervision costs, compared to 32% and 39% in Mozambique, respectively. Personnel (61%) were the largest share of supervision costs in Mozambique, comprised of salaries for implementing partner staff (99%) who carried out administrative and management tasks for the pilot study and local district supervisors (1%). Supervision in Ghana was conducted by salaried NMP, regional, and district health staff who received per diems for their travel (which were classified as services).

In terms of collection costs (Fig. 2), a larger share of Mozambique’s costs went to services (59%) compared to personnel (25%), reflecting the use of NMP staff for collections. By contrast, the share of personnel and service costs for collections in Ghana had similar proportions (44% and 36%, respectively). Only Mozambique incurred capital costs for collection activities (10%), which were spent on devices and accessories (e.g., laptop, tablet, battery chargers; Fig. 2).

**Fig. 3:**
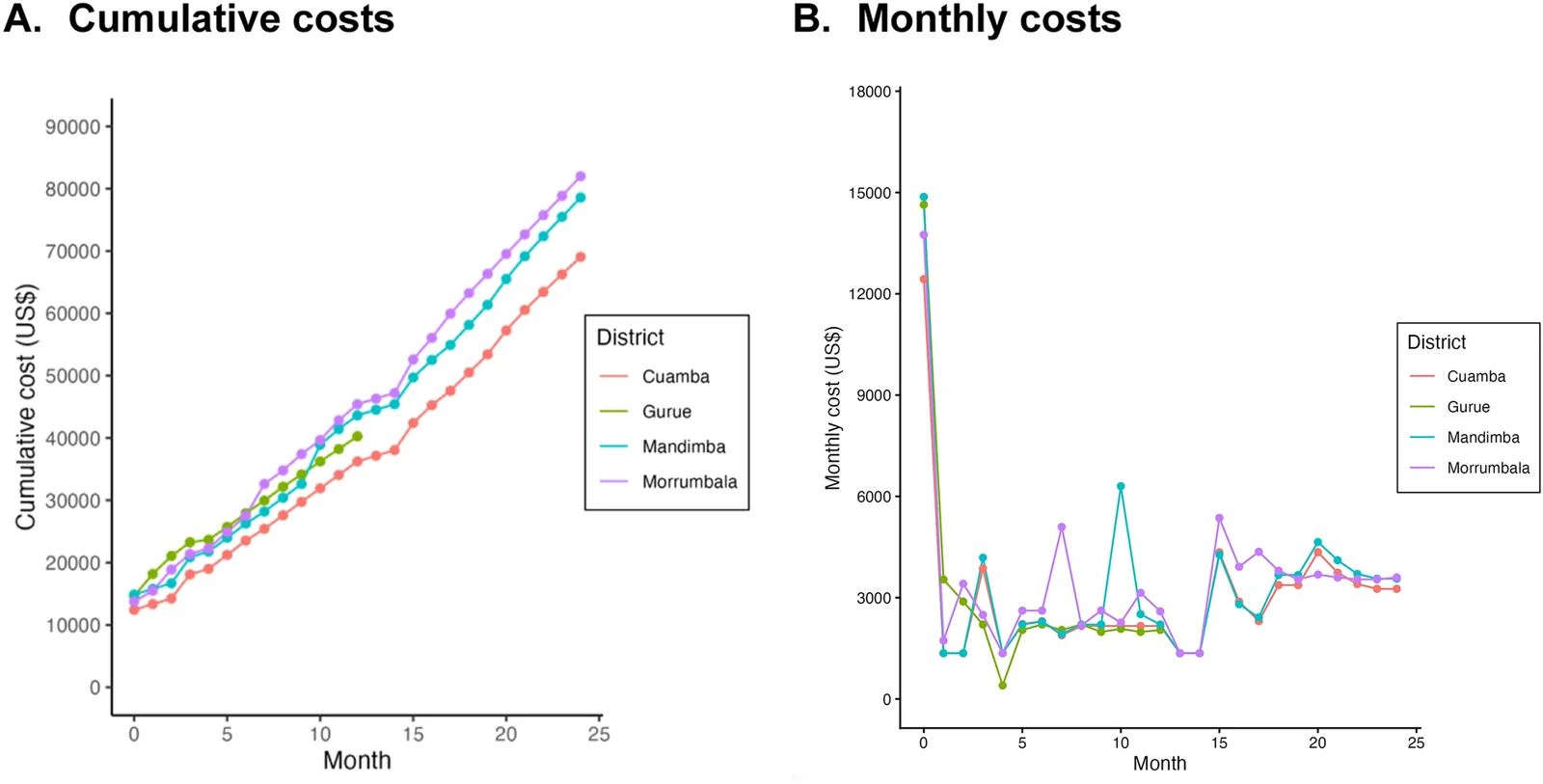
EASF costs over time by district in Mozambique. These line graphs show the cumulative (**A**) and per-month (**B**) costs of EASF implementation by district in Mozambique. *EASF* Entomological Adaptive Sampling Framework

**Fig. 4:**
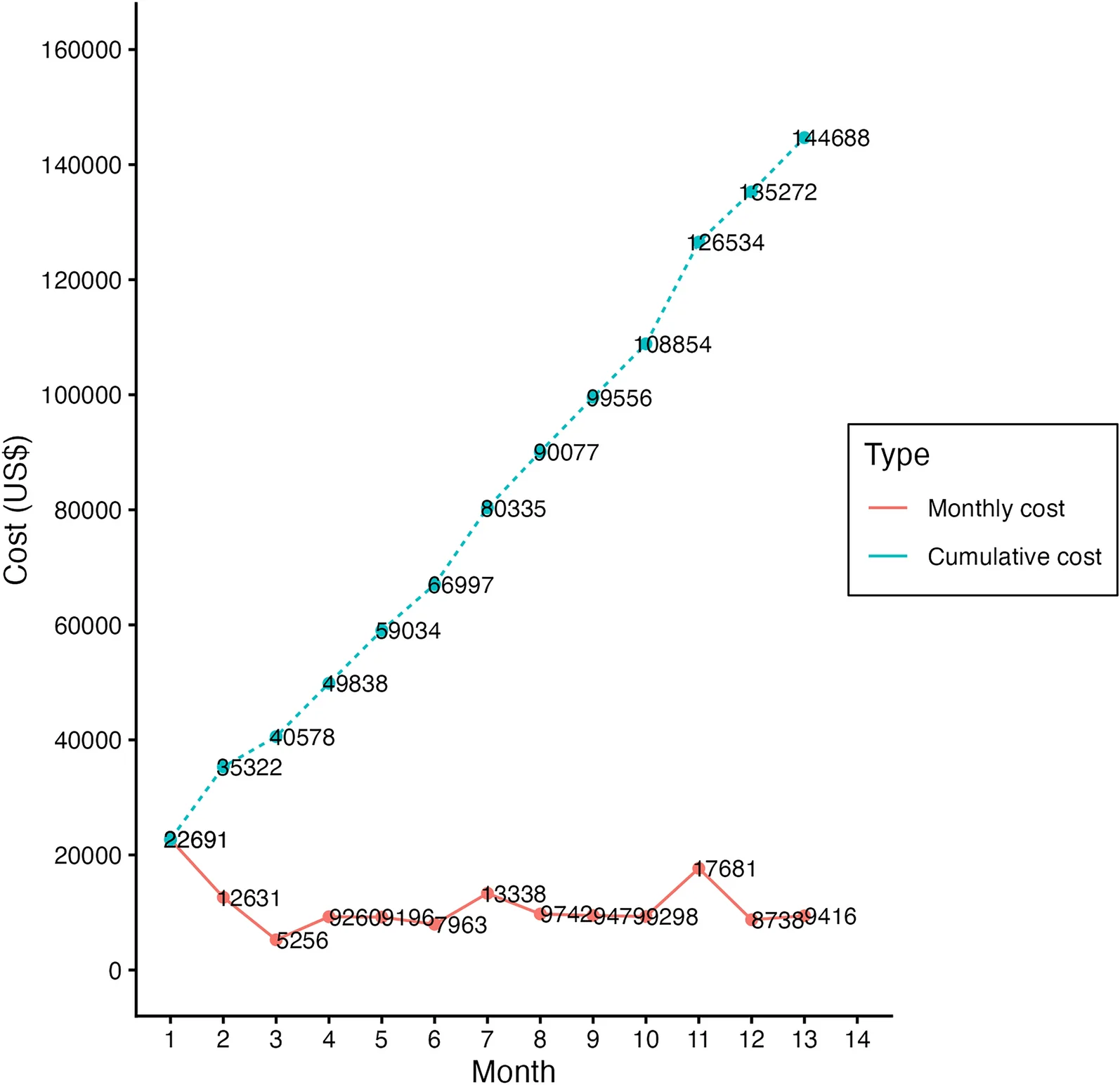
EASF costs over time in Ghana. These line graphs show the cumulative (blue line) and per-month (red line) costs of EASF implementation in Ghana across all districts. *EASF* Entomological Adaptive Sampling Framework

**Fig. 5:**
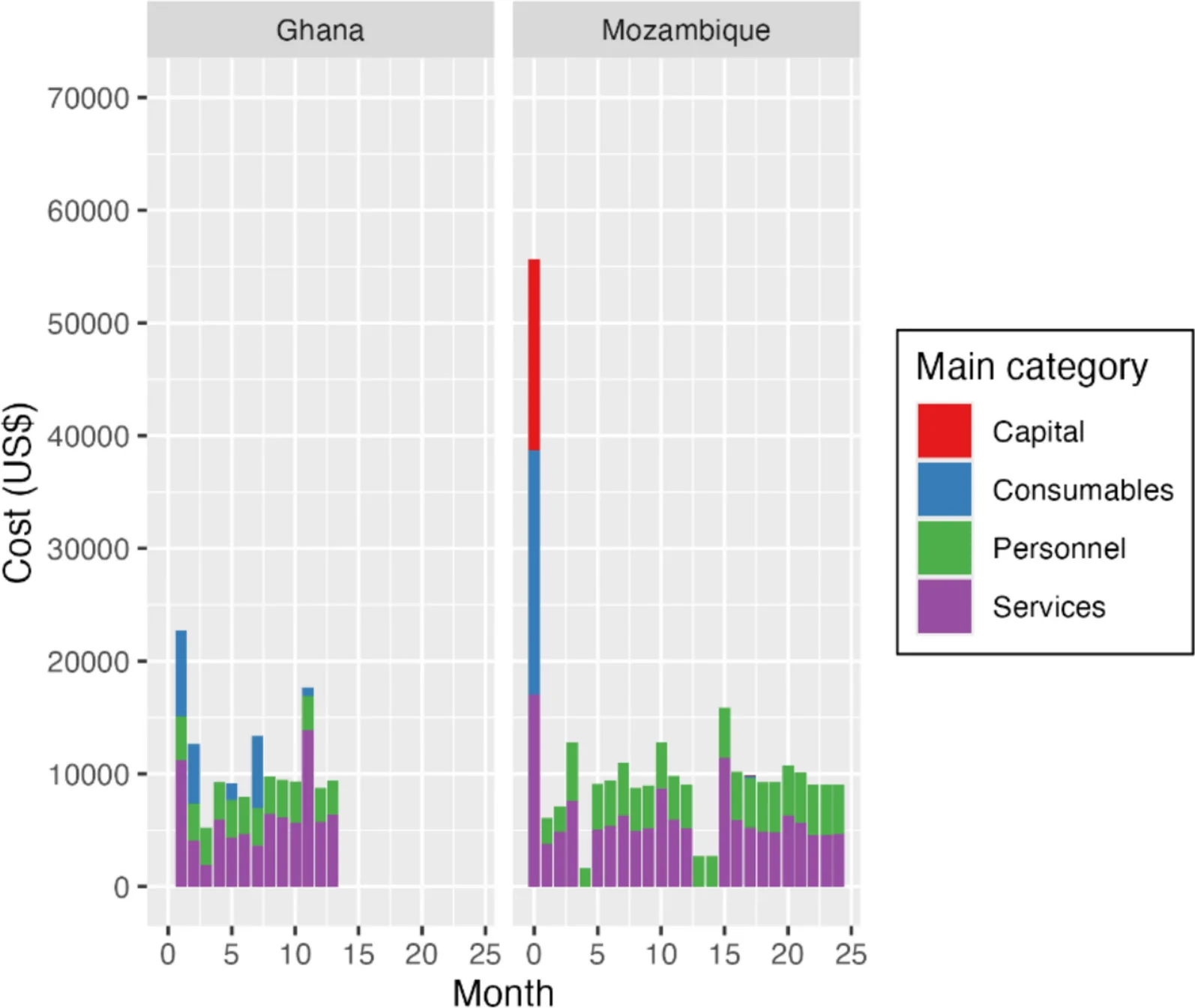
Monthly costs of EASF by category in Ghana and Mozambique. These bar charts show the monthly cost of EASF implementation by category (capital, consumables, personnel, and services) in Ghana (13 months) and Mozambique (24 months). *EASF* Entomological Adaptive Sampling Framework

The average cost per district was $28,938 (for 13 months) in Ghana and $66,476 (for 24 months) in Mozambique. The total costs per district in Mozambique, which represent different implementation timelines, are shown in Fig. 3 and Additional File 2. Excluding Gurué district, total EASF implementation costs were similar (range: $69,061–81,994; Fig. 3A) for the remaining 3 districts. While there were differences in monthly costs between districts in the early months of EASF implementation, costs stabilized and were similar starting in month 15 (Fig. 3B). Monthly costs in Ghana also varied by month (range: $5,256–22,691), with an average of $11,130 (Fig. 4). While consumables and capital costs were incurred primarily at the beginning of EASF implementation, personnel and services costs represented the majority of monthly costs in Ghana and Mozambique (Fig. 5).

### Cost analysis of EASF vs. routine entomological surveillance

**Fig. 6:**
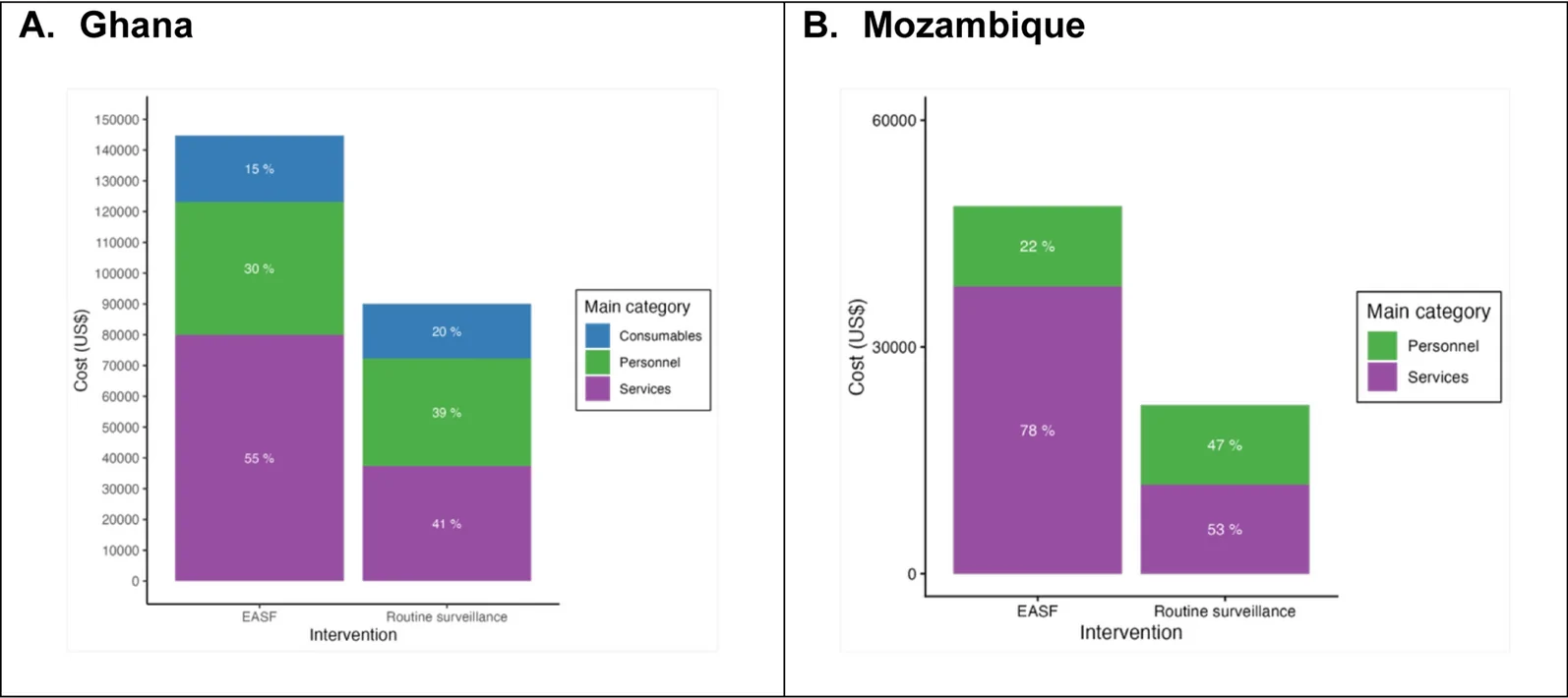
Incremental costs of EASF compared to routine entomological surveillance by category in Ghana and Mozambique. These bar charts show the total incremental cost of EASF compared to routine entomological surveillance by category (capital, consumables, personnel, and services) in Ghana (13 months) and Mozambique (9 months). *EASF* Entomological Adaptive Sampling Framework

Compared to routine entomological surveillance, EASF in Ghana was associated with $54,684 higher costs over 13 months of implementation ($144,688 vs. $90,004). In Mozambique, the incremental cost of EASF (in Cuamba and Mandimba only) was $26,335 over 9 months ($48,646 vs. $22,311). The share of main cost categories between the 2 interventions showed slight differences within and between countries (Fig. 6).

The unit costs of EASF and routine entomological surveillance per collection and per mosquito collected are shown in Table 3. In Ghana, EASF led to lower costs per collection ($48.29 vs. $157.35) and per mosquito ($11.10 vs. $43.65) compared to routine entomological surveillance. While EASF had higher costs per collection compared to routine entomological surveillance in Mozambique ($64.35 vs. $51.65), collections were more efficient, leading to lower costs per mosquito ($54.23 vs. $743.69).

**Table 3:** Unit costs of EASF and routine entomological surveillance in Ghana and Mozambique

Surveillance strategy	Ghana		Mozambique^a^	
	EASF	Routine surveillance	EASF	Routine surveillance
Effectiveness				
Collections	2996	572	756	432
Mosquitoes	13,036	2,062	897	30
Total costs (US$)	144,688	90,004	48,646	22,311
Unit costs (US$)				
Per collection	48.29	157.35	64.35	51.65
Per mosquito	11.10	43.65	54.23	743.69

^a^EASF costs are based on a standardized estimate for a 9-month period that overlaps with the period of data collection for routine entomological surveillance

### Cost-effectiveness of EASF vs. routine entomological surveillance

The cost-effectiveness results are summarized in Table 4. The ICERs per incremental mosquito collected were $4.98 in Ghana and $30.38 in Mozambique. The ICER per increase in the average number of mosquitoes per collection was $30.23 in Ghana and $72.76 in Mozambique.

**Table 4:** Incremental cost-effectiveness analysis of EASF compared to routine entomological surveillance in Ghana and Mozambique

Entomological surveillance strategy	Ghana		Mozambique^a^	
	Collections	Mosquitoes	Collections	Mosquitoes
Effectiveness				
EASF	2996	13,036	756	897
Routine	572	2,062	432	30
Incremental effectiveness	2424	10,974	324	867
Costs (US$)				
EASF	144,688		48,646	
Routine	90,004		22,311	
Incremental cost	54,684		26,335	
Incremental cost-effectiveness (US$)^c^				
Per mosquito collected	4.98		30.38	
Per increase in ave. mosquitoes collected^b^	30.23		72.76	

^a^EASF costs are based on a standardized estimate for a 9-month period that overlaps with the period of data collection for routine entomological surveillance. Effectiveness results are based on the outputs of EASF and routine entomological surveillance in Decemver 2023-August 2024 (9 months) only

^b^Calculated by dividing the incremental cost per collection by the incremental average mosquitoes per collection

^c^ICER calculations are based on unrounded raw data; minor discrepancies may occur when recalculating using the rounded values presented in the table

When the start-up costs of the EASF model ($75,000 per country) were included in the total costs of EASF, the ICER per mosquito collected increased to $11.82 in Ghana and $116.88 Mozambique. In terms of average mosquitoes per collection, the ICERs in Ghana and Mozambique increased to $71.63 and $279.99.

## Discussion

This study compared the cost and cost-effectiveness of EASF, a novel sampling strategy for malaria vector surveillance, with routine entomological surveillance in Ghana and Mozambique. Using data from pilot implementations, we found that EASF’s cost per collection was lower in Ghana compared to routine entomological surveillance, and the cost per mosquito collected was lower for EASF in both countries. In terms of cost-effectiveness, EASF’s incremental costs per mosquito collected were $4.98 and $30.38 in Ghana and Mozambique, respectively. For each increase in the average number of mosquitoes per collection in EASF, the costs were $30.23 and $72.76 in Ghana and Mozambique, respectively. As reported previously [3], EASF also outperformed routine entomological surveillance in other metrics, such as improving the quality (i.e., predictive capacity and accuracy) of surveillance data, after controlling for total sampling effort. Together, these results suggest that EASF may be a more efficient vector surveillance strategy compared to routine entomological surveillance.

Interpreting the ICERs we reported in this study may be limited. ICERs are typically compared to a cost-effectiveness threshold for a specific setting, which reflects a decision maker’s maximum willingness-to-pay or price for an additional unit of effectiveness, to determine whether an intervention is cost-effective or not. However, the cost-effectiveness threshold for entomological outputs like mosquitoes collected is not well-established. Instead, the health-economic literature has focused on estimating cost-effectiveness thresholds for ICERs that use health outcomes like disability-adjusted life years (DALYs) averted that can be used across different diseases and contexts [13, 14]. Assuming that an NMP’s willingness-to-pay for collections and mosquitoes is equal to the price it is currently paying—a form of “revealed price” [15]—then EASF may offer good economic value. For example, the cost per mosquito collected under routine surveillance in Ghana was $43.65, which is nearly 10 times higher than the incremental cost per mosquito collected for EASF ($4.98). Use of such thresholds, however, is limited because it relies on several assumptions and may be confounded by several unmeasured factors [16]. Additional research is needed to understand the maximum price NMPs are willing to pay for entomological outputs and activities.

Despite the extensive economic evaluation literature on malaria control and elimination interventions [17, 18], few have focused on entomological surveillance strategies. Prior studies have compared mosquito collection or trapping methods. For example, an analysis of community-based routine surveillance using Ifakara Tent Traps (ITTs) in Dar es Salaam found that, despite lower per-night sensitivity compared to HLC, the ITTs achieved comparable mosquito yields at a lower cost per mosquito collected (US$153 vs. US$187) [19]. Other studies have focused on the quality of vector collections conducted by different personnel. In western Kenya, unsupervised community-based collectors operated at much lower costs (US$9 vs. US$89 per person-night) than experienced entomology technicians. However, the community-based collectors captured fewer mosquitoes, highlighting a trade-off between quality and affordability in decentralized surveillance [20]. In a study in southeast Zambia, collections by community health workers were cheaper than collections done by NMP staff, whether they were measured in terms of cost per sampling night (e.g., US$14 vs. US$289 using CDC light traps) or per mosquito collected (e.g., US$5 vs. $141 using CDC light traps) [21]. These studies demonstrate that the value of novel entomological surveillance systems should be based on their costs and ability to generate sufficient, representative, and actionable entomological data.

Because of local contextual and implementation differences, comparing overall EASF costs between Ghana and Mozambique is difficult. Still, we found notable differences in cost structures: Ghana’s collection costs were split similarly between personnel and services, while in Mozambique, services—mainly per diems and transport—made up over half of collection costs. Mozambique also had capital expenses for collection, unlike Ghana. These contrasts underscore the importance of context‑specific cost assessments when planning national scale‑up.

A country’s decision to adopt EASF as a primary or supplemental entomological surveillance strategy will depend on several factors. The primary consideration is likely to be affordability. With declining donor and domestic malaria funding, NMPs may not have the fiscal space to continue historical levels of spending on entomological surveillance in the short term [22, 23]. Data from the WHO suggest that reductions in official development assistance in 2025 alone have led to moderate-to-severe disruptions to malaria services in 52% of WHO country offices [24]. In this analysis, we found evidence that EASF is more efficient at capturing mosquitoes than routine entomological surveillance, and it should therefore be considered as a viable option to maximize an NMP’s existing resources. To help countries make informed decisions about adopting or scaling up EASF, conducting a budget impact analysis can determine the financial implications of different implementation and financial (e.g., budget-neutral) scenarios. Such an analysis could bring to bear new efficiencies and/or economies of scale that may come with expanding EASF to more districts or provinces. For example, EASF uses the same sampling/trapping methods, personnel (e.g., entomology technicians), and workflows, and can be integrated without major structural changes. EASF can also be used to meet different goals, such as improving entomological data accuracy or reducing sampling efforts (i.e., increasing sampling yields), within operational and budget constraints. However, larger-scale and longer-term EASF implementation may require additional analytical capacity (in terms of computational infrastructure and trained analysts) to periodically update the model with new data and generate actionable outputs. This is evident in our sensitivity analysis, which found the ICER of EASF to increase by 2 or more times when the start-up costs of the EASF model were included.

The suitability of EASF will also depend on a country’s objectives for its entomological surveillance program. Our study suggests that EASF may lead to a higher number of mosquitoes caught, which may be especially important for low-transmission settings, settings where vector control strategies have reduced the vector population, or where live mosquitoes are needed for insecticide resistance bioassays [19, 21]. In settings where collections yield high numbers of mosquitoes, routine entomological surveillance efforts may suffice. A recent modeling study found that the expected value of perfect information, which represents the maximum value of investing in additional entomological data collection, was low (US$0.05–0.17 per person at risk), leading the authors to conclude that highly effective interventions like insecticide-treated bednets and indoor residual spraying should be implemented without delay, even when entomological data are incomplete [4]. Additional research should focus on quantifying the additional benefits of entomological surveillance data on understanding malaria transmission patterns and the ability of NMPs to allocate resources more cost-effectively.

### Limitations

The study has several limitations. Costs were measured during the pilot implementation period and may not reflect long-term or scaled-up implementation, where economies of scale could reduce unit costs. We excluded several costs, such as NMP personnel costs, which were assumed to be equal between EASF and routine entomological surveillance. However, the increased efficiency of entomological surveillance under EASF may lead to savings, including for personnel costs, which can be assessed through an operational or workload analysis.

We adopted a payer perspective, excluding any broader costs or benefits of EASF, such as staff time diverted from other responsibilities, opportunity costs to local health systems, or indirect benefits of improved data on malaria burden and control efforts. Data limitations narrowed the scope of our comparative analysis. For example, routine entomological surveillance costs in Mozambique were only collected in Year 2 for Mandimba and Cuamba districts. While the types of mosquito sampling (e.g., HLC, PSC) were standardized between EASF and routine entomological surveillance within countries, the distribution of sampling methods varied between the two strategies, which may have contributed to differences in costs and effectiveness. Finally, we measured effectiveness in terms of entomological outputs (collections and mosquitoes collected) rather than epidemiological or health outcomes such as malaria cases or DALYs averted, as recommended in economic evaluations. Entomological data generated by surveillance strategies like EASF are critical for NMP decision-making and resource allocation, which ultimately affect malaria transmission in the community. However, empirical research linking entomological surveillance strategies such as EASF to downstream health outcomes remains limited and should be the focus of future research, including simulation studies.

## Conclusion

Our economic evaluation found that EASF may be a cost-effective approach to entomological surveillance in malaria-endemic settings. Despite higher overall costs compared to routine entomological surveillance, EASF’s adaptive design yields substantially greater entomological outputs at lower unit costs, which may provide additional actionable information for NMPs. Future research should assess the long-term costs, epidemiological benefits, and implementation challenges of EASF across diverse settings to fully capture its potential value.

## Supplementary Information

Supplementary Material 1.

## Data Availability

The datasets used and/or analysed during the current study are available from the corresponding author on reasonable request.

## Abbreviations

CDC: Centers for Disease Control and Prevention
DALY: Disability-adjusted life year
EASF: Entomological Adaptive Sampling Framework
HLC: Human landing catch
ICER: Incremental cost-effectiveness ratio
ITT: Ifakara Tent Trap
LT: Light trap
NMP: National malaria program
PSC: Pyrethrum spray catch
WHO: World Health Organization

## References

[1] World Health Organization. Malaria surveillance, monitoring & evaluation: a reference manual. World Health Organization; 2018.

[2] Mayor A, Ishengoma DS, Proctor JL, Verity R. Sampling for malaria molecular surveillance. Trends Parasitol. 2023;39(11):954–68. 10.1016/j.pt.2023.08.007. PMC10580323 37730525

[3] Sedda L, Jalilian A, Opiyo M, et al. Evaluation of the entomological adaptive surveillance framework for malaria vector monitoring: a comparative trial with routine surveillance in Ghana and Mozambique. *VeriXiv*. 2025. https://verixiv.org/articles/2-331.Accessed 10 Nov 2025. 10.1136/bmjph-2025-004060 PMC13052570 41948179

[4] Schmit N, Topazian HM, Pianella M, et al. Quantifying the potential value of entomological data collection for programmatic decision-making on malaria control in sub-Saharan African settings. Malar J. 2025;24(1):31. 10.1186/s12936-025-05251-7. PMC11780794 39885514

[5] van de Straat B, Russell TL, Staunton KM, Sinka ME, Burkot TR. A global assessment of surveillance methods for dominant malaria vectors. Sci Rep. 2021;11(1):15337. 10.1038/s41598-021-94656-w. PMC8319300 34321525

[6] Mawejje HD, Asiimwe JR, Kyagamba P, et al. Impact of different mosquito collection methods on indicators of Anopheles malaria vectors in Uganda. Malar J. 2022;21:388. 10.1186/s12936-022-04413-1. PMC9761930 36536428

[7] Kouassi BL, Edi C, Ouattara AF, et al. Entomological monitoring data driving decision-making for appropriate and sustainable malaria vector control in Côte d’Ivoire. Malar J. 2023;22(1):14. 10.1186/s12936-023-04439-z. PMC9835745 36635720

[8] Husereau D, Drummond M, Augustovski F, et al. Consolidated health economic evaluation reporting standards (CHEERS) 2022 explanation and elaboration: a report of the ISPOR CHEERS II good practices task force. Value Health. 2022;25(1):10–31. 10.1016/j.jval.2021.10.008. 35031088

[9] Vassall A, Sweeney S, Kahn JG, et al. Reference case for estimating the costs of global health services and interventions. 2017. https://researchonline.lshtm.ac.uk/id/eprint/4653001/1/vassall_etal_2018_reference_case_for_estimating_costs_global_health_services.pdf. Accessed 26 June 2025.

[10] NICE International. The gates reference case: what it is, why it’s important, and how to use it. 2014. https://www.idsihealth.org/wp-content/uploads/2016/05/Gates-Reference-case-what-it-is-how-to-use-it.pdf. Accessed 26 June 2025.

[11] Špacírová Z, Epstein D, García-Mochón L, Rovira J, Olry de Labry Lima A, Espín J. A general framework for classifying costing methods for economic evaluation of health care. Eur J Health Econ. 2020;21(4):529–42. 10.1007/s10198-019-01157-9. PMC8149350 31960181

[12] Hutubessy R, Chisholm D, Edejer TTT. Generalized cost-effectiveness analysis for national-level priority-setting in the health sector. Cost Eff Resour Alloc. 2003;1:8. 10.1186/1478-7547-1-8. PMC320499 14687420

[13] Revill P, Ochalek J, Lomas J, et al. Cost-effectiveness thresholds: guiding health care spending for population health improvement. In: World Scientific Series in Global Health Economics and Public Policy. World Scientific. 2020;05:75–97. 10.1142/9789813272378_0003.

[14] Chi YL, Blecher M, Chalkidou K, et al. What next after GDP-based cost-effectiveness thresholds? Gates Open Res. 2020;4:176. 10.12688/gatesopenres.13201.1. PMC7851575 33575544

[15] Deb R, Kitamura Y, Quah JKH, Stoye J. Revealed price preference: theory and empirical analysis. Rev Econ Stud. 2023;90(2):707–43. 10.1093/restud/rdac041.

[16] Tipoe E, Adams A, Crawford I. Revealed preference analysis and bounded rationality. Oxf Econ Pap. 2022;74(2):313–32. 10.1093/oep/gpab018.

[17] Shretta R, Avanceña ALV, Hatefi A. The economics of malaria control and elimination: a systematic review. Malar J. 2016;15(1):593. 10.1186/s12936-016-1635-5. PMC5154116 27955665

[18] Conteh L, Shuford K, Agboraw E, Kont M, Kolaczinski J, Patouillard E. Costs and cost-effectiveness of malaria control interventions: a systematic literature review. Value Health. 2021;24(8):1213–22. 10.1016/j.jval.2021.01.013. PMC8324482 34372987

[19] Chaki PP, Mlacha Y, Msellemu D, et al. An affordable, quality-assured community-based system for high-resolution entomological surveillance of vector mosquitoes that reflects human malaria infection risk patterns. Malar J. 2012;11(1):172. 10.1186/1475-2875-11-172. PMC3475008 22624853

[20] Abong’o B, Stanton MC, Donnelly MJ, et al. Evaluation of community-based vector surveillance system for routine entomological monitoring under low malaria vector densities and high bed net coverage in western Kenya. Malar J. 2023;22(1):203. 10.1186/s12936-023-04629-9. PMC10318638 37400805

[21] Sikaala CH, Chinula D, Chanda J, et al. A cost-effective, community-based, mosquito-trapping scheme that captures spatial and temporal heterogeneities of malaria transmission in rural Zambia. Malar J. 2014;13(1):225. 10.1186/1475-2875-13-225. PMC4060139 24906704

[22] Rannan-Eliya RP. Financing malaria. PLOS Global Public Health. 2022;2(6):e0000609. 10.1371/journal.pgph.0000609. PMC10021190 36962472

[23] RBM Partnership. Funding landscape. 2025. https://dashboards.endmalaria.org/en/funding-landscape. Accessed 20 Aug 2025.

[24] World Health Organization. The impact of suspensions and reductions in health official development assistance on health systems. 2025. https://www.who.int/publications/m/item/the-impact-of-suspensions-and-reductions-in-health-official-development-assistance-on-health-systems. Accessed 20 Aug 2025.

