# Supplementary material for: Entomological Adaptive Sampling Framework (EASF) for malaria vector surveillance: an economic evaluation of pilot implementation in Ghana and Mozambique
Source: Malar J. 2026 Jul 13;25:359. doi: 10.1186/s12936-026-06020-w (PMC13647601; doi:10.1186/s12936-026-06020-w)
Supplement: Supplementary file 1 [file 12936_2026_6020_MOESM1_ESM.docx]

# SUPPLEMENTary information

## Additional file 1. Timeline of EASF pilot implementation in Ghana and Mozambique

EASF, Entomological Adaptive Sampling Framework.

## Additional file 2. Total cost of EASF implementation by district in Mozambique

| **District** | **Cost (US$)** | **Percent (%) of total** |
| --- | --- | --- |
| Gurué^a^ | 40,264 | 15 |
| Cuamba^b^ | 69,061 | 26 |
| Mandimba^b^ | 78,584 | 29 |
| Morrumbala^c^ | 81,994 | 30 |

^a^Sep 2022-Aug 2023

^b^Jan 2023-Aug 2024

^c^Oct 2022-Aug 2024
